# Coexisting Cyclic Parthenogens Comprise a Holocene Species Flock in *Eubosmina*

**DOI:** 10.1371/journal.pone.0011623

**Published:** 2010-07-16

**Authors:** Markéta Faustová, Veronika Sacherová, H. David Sheets, Jan-Erik Svensson, Derek J. Taylor

**Affiliations:** 1 Department of Biological Sciences, State University of New York at Buffalo, Buffalo, New York, United States of America; 2 Department of Ecology, Charles University, Prague, Czech Republic; 3 Department of Physics, Canisius College, Buffalo, New York, United States of America; 4 School of Engineering, University College of Borås, Borås, Sweden; 5 Medins Biology AB, Mölnlycke, Sweden; Institute of Evolutionary Biology (CSIC-UPF), Spain

## Abstract

**Background:**

Mixed breeding systems with extended clonal phases and weak sexual recruitment are widespread in nature but often thought to impede the formation of discrete evolutionary clusters. Thus, cyclic parthenogens, such as cladocerans and rotifers, could be predisposed to “species problems” and a lack of discrete species. However, species flocks have been proposed for one cladoceran group, *Eubosmina*, where putative species are sympatric, and there is a detailed paleolimnological record indicating a Holocene age. These factors make the *Eubosmina* system suitable for testing the hypotheses that extended clonal phases and weak sexual recruitment inhibit speciation. Although common garden experiments have revealed a genetic component to the morphotypic variation, the evolutionary significance of the morphotypes remains controversial.

**Methodology/Principal Findings:**

In the present study, we tested the hypothesis of a single polymorphic species (i.e., mixing occurs but selection maintains genes for morphology) in four northern European lakes where the morphotypes coexist. Our evidence is based on nuclear DNA sequence, mitochondrial DNA sequence, and morphometric analysis of coexisting morphotypes. We found significant genetic differentiation, genealogical exclusivity, and morphometric differentiation for coexisting morphotypes.

**Conclusions:**

We conclude that the studied morphotypes represent a group of young species undergoing speciation with apparent reproductive barriers despite coexistence in the freshwater pelagic zone.

## Introduction

Breeding systems are expected to play a central role in speciation [1]–[4]. In strictly sexual species, gene flow can unite populations, whereas ecological divergence and a lack of interbreeding can result in the formation of discrete evolutionary clusters. Likewise, in strictly asexual species, periodic selective sweeps of clones may unite populations and discontinuous ecological niches might lead to the appearance of discrete evolutionary clusters [5]. However, in groups with mostly asexual systems, extended clonal phases followed by weak sexual recruitment may prevent the formation of discrete lineages. In these mixed breeding systems recombination might be sufficient to prevent clonal selective sweeps but insufficient to homogenize populations by gene flow [2], [6]. In addition, mixed breeding systems are often associated with small organisms and powerful dispersal abilities, potentially reducing the capacity for speciation [1], [4]. Mixed breeding systems might also promote the formation (sexual phase) and stabilization (clonal phase) of hybrid products, further blurring the boundaries of evolutionary clusters. Finally, experimental results indicate that animals with mixed breeding systems suffer a reduced rate of adaptation compared to those with obligate outcrossing [7]. Thus, organisms with mixed breeding systems such as cyclic parthenogens (cladocerans, rotifers, aphids etc.) could be predisposed to slow rates of cladogenesis, and lack rapid radiations.

An opposing view is that mixed breeding systems, such as cyclical parthenogenesis, can imbue organisms with a capacity for quantum phenotypic evolution and potentially enhance speciation rates [8], [9]. Here, the action of mutation and selection on multiple rounds of asexual reproduction results in a build up of unexpressed genetic variance. Infrequent sexual reproduction can then cause a flush of expressed genetic variance and enhanced capacity for adaptation. For cyclic parthenogens several rapid radiations have been proposed for allopatric forms, and there is indirect evidence for their rapidity [10]. However, only one group of lacustrine cladocerans, the *Eubosmina* group has an excellent continuous paleolimnological record of a radiation during the Holocene [11]–[28] and proposed sister taxa coexist in the same lakes. The recent emergence of the morphotypes is well documented in the subfossil record; only the *longispina* morphotype has been recorded from the interglacial and late glacial sediments - all other morphotypes are restricted to postglacial sediments. Eleven taxa are recognized in the most recent taxonomic treatment of the complex [29] but whether morphotypes represent real evolutionary lineages or merely polymorphisms remains a controversy [30]. The taxa are diagnosed by the shapes of the carapace, antennules, and paired posterior spines called mucros. *Eubosmina* are unique, among freshwater cladocerans, in the existence of putative species flocks - up to nine taxa can co-occur in the same lake [31]. Phenotypic plasticity is evident in the diagnostic characters [32], [33], but common garden experiments have revealed that the morphological differences have a genetic component. That is, clones from different taxa cultured under identical conditions retain some of their characteristic morphology [34].

Ecological differentiation among *Eubosmina* morphotypes is most pronounced between *E. longispina*, which is usually found in oligotrophic waters, and the extreme forms e.g. *berolinensis*, *gibbera* and *thersites*, which are usually found in more eutrophic lakes. The morphological differences in antennules, brood chambers and posterior spines have been found to reduce vulnerability to invertebrate predation [34]–[38]. Thus, abundances of the extreme forms are correlated with invertebrate predators and the nutrient status of lakes seasonally, spatially, and temporally [18], [39]–[42].

A critical evolutionary question remains: do the coexisting morphotypes of *Eubosmina* represent polymorphisms or discrete evolutionary lineages? The evolutionary status of the morphotypes of *Eubosmina* is a longstanding controversy [29], [30], [34], [43]. Earlier genetic analysis using allozymes [34], and sequence variation in the nuclear rDNA array (partial *18S*, *ITS*, partial *28S*) and *16S* mtrDNA regions [30] found that the European morphs were closely related, but were unable to rule out random morphotype-genotype associations. Hellsten & Sundberg [44] reported genetic isolation based on non-statistical patterns (multidimensional scaling) of RAPD markers between coexisting *E. longispina* and *E. coregoni* in Östersjön Lake, Sweden. However, they also reported that over 15% of the RAPD bands were not repeatable and the contribution of non-cladoceran DNA (microbes, algae, symbionts) to the patterns with anonymous RAPD markers is unknown. Here, we explicitly test the hypothesis of evolutionary discreteness in the *Eubosmina* group using nuclear and mitochondrial DNA sequences and geometric morphometric analysis from the same individuals. We chose four lakes that contain different combinations of coexisting morphotypes. Testing for reproductive barriers in coexisting populations (rather than among allopatric populations) is straightforward but important because a small amount of interbreeding should homogenize coexisting lineages. Significant morphometric and genetic differentiation found in coexisting taxa is consistent with reproductive barriers but inconsistent with geographic isolation. Reciprocal monophyly is neither expected nor observed in young radiations [45]–[49]. If the morphotypes do represent young (postglacial) lineages, then we expect the genetic patterns found in haplotype networks to have the characteristic signature of young lineages. That is, we expect some sharing of haplotypes among coexisting species and low but significant measures of genetic divergence. Often presumed older central haplotypes are shared among recently diverged species [45], [46], [49]. Genetic divergence is therefore a continuum that proceeds with time from differentiation with sharing to monophyly. Reduced sharing of haplotypes, significant genetic divergence, and significant morphometric differentiation of coexisting forms are evidence of reproductive barriers.

## Results

### Genetic Analyses

**Figure 1:**
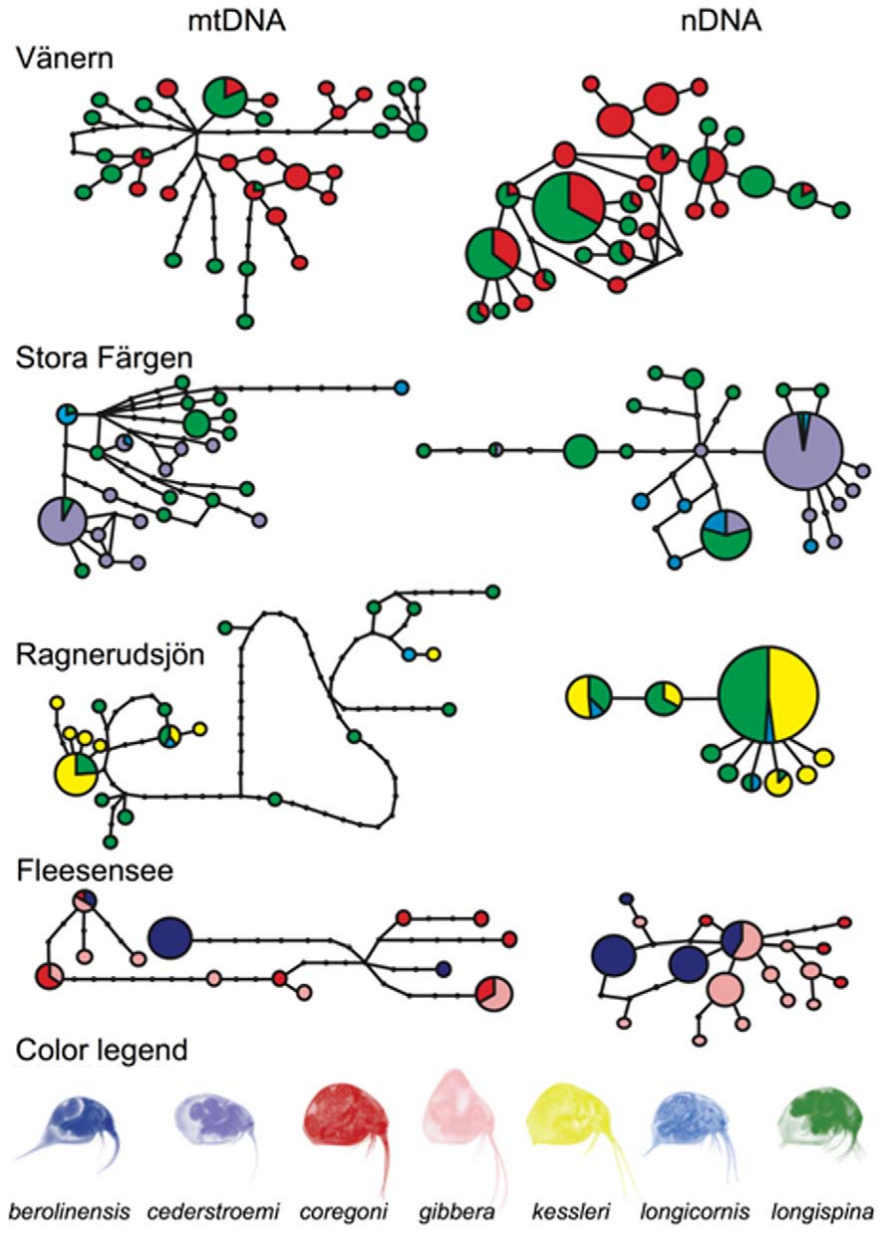
TCS networks of *ND2* and *HSP* haplotypes. *ND2* haplotypes in the left column and the *HSP* haplotypes in the right column, representing haplotypes of specimens from lakes (from the top to the bottom): Vänern, Stora Färgen, Ragnerudsjön and Fleesensee. Area of circle is proportional to the number of individuals sharing the haplotype. Small, uncolored circles represent missing intermediate haplotypes. Circles with following numbers of individuals sharing he haplotype were suggested as central/ancestral haplotypes (from the top to the bottom): 20, 24, 18, 5 for ND2 and 5 (next 24), 43, 12, 15 for HSP. Colors correspond to different morphotypes: *coregoni*-red; *longispina*-green, *cederstroemi*-violet; *longicornis*-light blue; *kessleri*-yellow; *berolinensis*-dark blue; *gibbera*-pink; and are proportional to the numbers of specimens of the morphotypes sharing one haplotype. Apparent non random- low sharing of haplotypes among/between morphotypes reflects their significant genetic separation in amount expected for young species undergoing speciation.

Genetic differentiation was significant by most measures for coexisting morphotypes of *Eubosmina* (Table 1). Both *ND2* and *HSP90* showed significant genetic differentiation among coexisting morphs with the exception of those from Ragnerudsjön Lake. The mitochondrial gene *ND2* exhibited less differentiation than the nuclear gene *HSP90* analyses. FST values differed significantly from zero in all the cases for *ND2* and for six out of seven comparisons for *HSP90*. Sequential ΦST values differed significantly from zero in nine out of eleven cases for *ND2* and in eight of eleven cases for *HSP90* haplotypes (Table 1). For individual lakes, both FST and ΦST values were significantly greater than zero for both analyzed genes of *longispina* and *coregoni* from Vänern Lake, and *longispina*, *cederstroemi* and *longicornis* from Stora Färgen Lake. FST values for *ND2* and *HSP90* and ΦST for *HSP90* of *berolinensis*, *coregoni*, and *gibbera* from Fleesensee Lake were significantly greater then zero as well. The ΦST for *HSP90* (*coregoni* and *gibbera*) was, however, not significant. For Ragnerudsjön Lake, only FST and ΦST values of *ND2* gene haplotypes belonging to *longispina* and *kessleri* and ΦST value of *ND2* comparing *longicornis* and *kessleri* haplotypes were significantly higher than zero. Inspection of TCS haplotype networks (Fig. 1) revealed that morphotypes often shared the most common and interconnected haplotypes (the presumed ancestral haplotypes), but had several private haplotypes (the exception was *HSP90* from Ragnerudsjön Lake (Fig. 1). Only 3 of 33 *ND2* haplotypes and 9 of 28 *HSP90* haplotypes were shared between *longispina* and *coregoni* from Vänern Lake. 3 of 25 *ND2* haplotypes and 3 of 22 *HSP90* haplotypes belonging to *longispina*, *cederstroemi* and *longicornis* from Stora Färgen were shared. For Ragnerudsjön Lake, mtDNA showed significant differentiation as only 3 of 21 *ND2* haplotypes were shared, nDNA haplotypes lacked differentiation as there were only four unique haplotypes from a total of nine closely related haplotypes. Also 3 of 13 *ND2* haplotypes and 1 of 18 *HSP90* haplotypes from *berolinensis*, *coregoni*, *gibbera* and *berolinensis* and *gibbera* morphotypes, respectively, were shared in the Fleesensee. Although we lack sequences of both genes for all of the analyzed specimens, it is obvious from our data that most individuals possessed morphotype-specific combined *ND2-HSP90* haplotypes. Genealogical sorting index values (*gsi*) indicated that morphotypes within all lakes but Ragnerudsjön showed significant lineage divergence (Table 1). Among the three lakes with significant sorting only the *HSP90* locus for *longispina* had a non-significant value.

**Table 1:** Pairwise FST, Φ_ST_, genealogical sorting index (*gsi*), Goodall's F test, Procrustes distances and correct jackknife grouping of morphotypes within each lake.

Lake	Morphotypes		FST		ΦST		GSI		Goodall's F test		Procrustes distance	Correct jackknife assignments (%)
	(*ND2*; *HSP*)	(*ND2*; *HSP*)	*ND2*	*HSP*	*ND2*	*HSP*	*ND2*	*HSP*	F	p		
Vänern	*longispina*	*coregoni*	**0.76**	**0.03**	**0.14**	**0.08**	GSI_l_ = **0.267**	GSI_l_ = 0.067	**150.7**	p<0.001	0.13	97.73
	(39; 41)	(40; 40)					GSI_c_ = **0.200**	GSI_c_ = **0.241**				
Stora Färgen	*longispina*	*cederstroemi*	**0.62**	**0.37**	**0.31**	**0.46**	GSI_l_ = **0.701**	GSI_l_ = **0.438**	**187.4**	p<0.001	0.12	91.67
	(26; 22)	(35; 27)					GSI_c_ = **0.546**	GSI_c_ = **0.411**				
	*longispina*	*longicornis*	**0.66**	**0.06**	**0.19**	**0.15**	x	x	**13.74**	p<0.001	0.05	
	(26; 22)	(7; 7)					x					
	*cederstroemi*	*longicornis*	**0.44**	**0.38**	**0.46**	**0.52**	x	x	**48.67**	p<0.001	0.07	
	(35; 27)	(7; 7)					x					
Ragnerudsjön	*longispina*	*kessleri*	**0.47**	0.003	**0.16**	0.004	GSI_l_ = 0.079	GSI_l_ = 0.050	**19.77**	p<0.001	0.04	53.33
	(19; 23)	(23; 26)					GSI_k_ = 0.140	GSI_k_ = 0.072				
	*longispina*	*longicornis*	x	x	0.07	0.08	x	x	1.19	p = 0.316	0.02	
	(19; 23)	(2; 3)					x					
	*longicornis*	*kessleri*	x	x	**0.51**	0.06	x	x	**4.17**	p = 0.017	0.03	
	(2; 3)	(23; 26)					x					
Fleesensee	*berolinensis*	*coregoni*	**0.55**	x	**0.52**	**0.28**	x	x	**5.33**	p = 0.014	0.04	94.12
	(19; 18)	(13; 2)					x					
	*berolinensis*	*gibbera*	**0.57**	**0.18**	**0.54**	**0.31**	GSI_b_ = **0.500**	GSI_b_ = **0.416**	**14.37**	p<0.001	0.09	
	(19; 18)	(17; 18)					GSI_g_ = **0.640**	GSI_g_ = **0.676**				
	*coregoni*	*gibbera*	**0.28**	x	0.01	**0.16**	x		**8.08**	p<0.001	0.05	
	(13; 2)	(17; 18)					x					

Statistically significant values (P<0.05) are shown in bold. x – values not calculated because of uneven or small sample sizes. Numbers in brackets below the morphotype names represent the counts of analysed specimens for *ND2* and *HSP* genes.

### Morphometric Analyses

**Figure 2:**
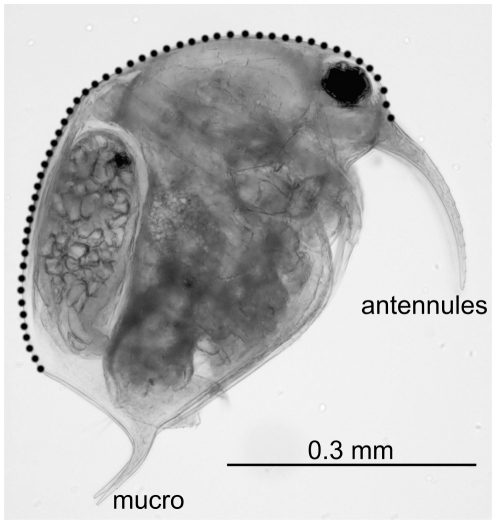
*Eubosmina* specimens. Doted line shows the dorsal margin of the carapax used for geometric morphometric comparisons.

The morphometric analysis (of size-free shape variation) mirrored the genetic analysis. Morphometric analyses of dorsal margins (Fig. 2) supported the discreteness of nearly all co-existing morphotypes. Goodall's F test showed significant differences of the mean carapace shapes relative to within group variances in nine of ten comparisons (Table 1). The sole non-significant value involved the *longispina* and *longicornis* morphs from Ragnerudsjön Lake (F: 1.19, p< = 0.316).

**Figure 3:**
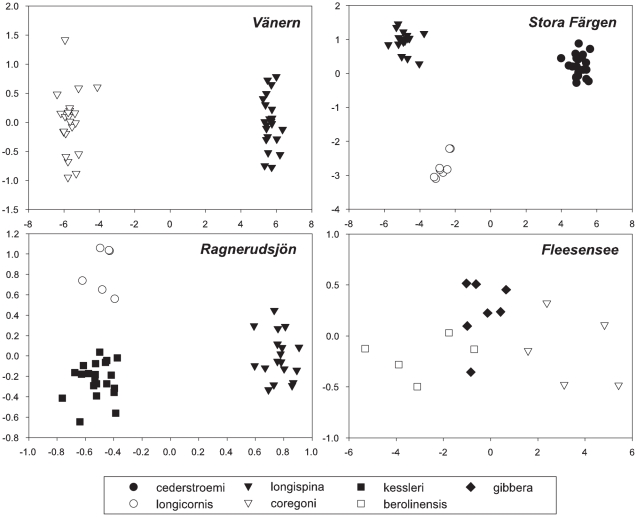
The CVA axis plots revealing morphometric discretness of carapace shape among the coexisting morphotypes. The X-axis reveals significant differentiation of carapace shape purely among morphotypes in each plot. The Y-axis reveals significant differentiation only for Stora Färgen Lake. CVA scores were transformed by a factor of 100 for Fleesensee Lake and 10,000 for the other lakes to improve visualization of the axis labels. The morphotype symbols are provided in the legend.

The CVA plot (Fig. 3) revealed the discreteness of the morphotypes in each lake with the first axis. The second axis showed separation only for the morphotypes of Stora Färgen Lake. The weakest differentiation in mean shapes was found in Ragnerudsjön Lake (Table 1).

Assignment analyses using jackknifed CVA assignments supported the discreteness of most morphotypes as assignment probabilities were greater than 91% in Vänern, Stora Färgen and Fleesensee Lakes (Table 1). The degree of differentiation among morphotypes within a lake resulted in 43 correct assignments and 1 incorrect assignment in Vänern Lake, 44 correct assignments and 4 incorrect assignments in Stora Färgen Lake, and in 16 correct assignments and 1 incorrect assignment in Fleesensee Lake. The *longispina*, *kessleri* and *longicornis* carapace morphotypes from Ragnerudsjön Lake could not be effectively discriminated - jackknifed groupings produced 24 correct and 21 incorrect assignments.

## Discussion

The statistically significant genetic and morphological differentiation of co-existing morphotypes of *Eubosmina* is inconsistent with the hypothesis of simple polymorphisms. Instead, because even very weak gene flow will homogenize coexisting taxa, the evidence supports the existence of reproductive barriers. The nature of the isolating mechanisms for the *Eubosmina* is unknown but taken together with existing paleolimnological, biogeographical, and ecological evidence, our results provide empirical evidence that animals with a mixed sexual and asexual breeding system are susceptible to the fastest of radiations. The diversification rate of *Eubosmina* is on par with another cyclic parthenogen, the pea aphid, that was estimated to have among the fastest diversification rates known for animals at 1 divergence event every 6,700 years [10]. If there are at least 10 lineages originating during the Holocene (12,000 years), then we estimate a minimum of 1 divergence event every 5, 212 years for *Eubosmina* [2], [10].

Although the genetic and morphometric evidence is consistent with the existence of reproductive barriers among coexisting morphotypes, occasional gene flow or introgression cannot be ruled out. Some proposed vertebrate radiations have been challenged on the grounds that hybridization is common, long-term continuity of the morphotypes is unlikely, and genealogical exclusivity is absent [50]–[52]. In the present case, continuity and coexistence of the morphotypes of *Eubosmina* for thousands of years is well documented in the paleolimnological record of many European lakes. We have shown that significant but not complete genealogical exclusivity is present among sympatric *Eubosmina*. Complete genealogical exclusivity is, of course, unexpected for Holocene-scale differentiation in animals. We observed the expected pattern of genetic differentiation with incompletely sorted lineages [45], [46], [49], [53]–[55]. Significant genetic and morphological differentiation among co-existing morphotypes is consistent with a reproductive barrier (and difficult to explain by another process). The differentiation in genotype frequencies, morphology, and the continuous sediment record of coexistence in some lakes is undiminished by the expected existence of shared haplotypes. Indeed, denial of the existence of young species based on the presence of shared alleles precludes the study of recent adaptive radiations.

There are however, some lakes in which apparent intermediate morphotypes of *Eubosmina* emerge [17], [18], [22]–[24]. In one of the four lakes (Ragnerudsjön), morphometrics failed to clearly assign morphotypes based on carapace shape – and this was the sole lake that lacked significant lineage differentiation (*gsi*) and genetic differentiation. The failure of the analysis in this lake could be due to the presence of intermediate forms between *longispina* and *kessleri* in Ragnerudsjön or simply a relatively recent divergence. Most of these “intermediates” are assigned to *longicornis* morphotype in this study. A larger sampling of the genomes is necessary to determine if there is introgression beyond early generation hybrids. Hybridization among cladoceran lineages is well known, but most and often leads to a markedly reduced clonal diversity in the hybrid lineages compared to the parental lineages [56]–[58]. This pattern is clearly absent in the current study as coexisting morphotypes share similar haplotype diversity. We note that approximately 75% of the adult individuals in Ragnerudsjön can be distinguished using the shape and the size of antennules and mucros – characters that we excluded because they are used to define most morphotypes. Overall, our geometric morphometric analyses confirm the existence of distinct morphotypes within *Eubosmina* in all four lakes with some intermediates in Ragnerudsjön Lake. We conclude that when antennules and mucro shapes are included, identification according to Lieder's system [29] can be safely applied.

There are several converging lines of evidence to support the very young age of the *Eubosmina* radiation. The detailed paleolimnological record of morphotype evolution from many lakes agrees on a postglacial origin for all morphotypes but *longispina*. Most morphotypes are restricted to non-refugial lakes that are less than 20,000 years old. Biogeographic evidence and the association of morphotypes with cultural eutrophication also support a recent radiation. Finally, the patterns of haplotype divergence found in our study are also consistent with recent lineage formation, sharing is largely restricted to the more common central haplotypes. Further evidence of recent radiation could be provided by coalescent analyses, but the strong possibility of numerous parallel origins of morphotypes that are apparent from some of haplotype networks (Fig. 1) and in paleolimnological records complicates an approach that depends on lineages having the same common ancestor. The increased complexity of our observed networks from more compact starlike networks with one evident central haplotype [45], [49] may be attributable to multiple origins of morphotypes.

If radiations in organisms with mixed breeding systems can be rapid, then why are so few known? Most organisms with mixed breeding systems are very small and lack a detailed fossil record. Studies of species diversity, the tempo of evolution, and morphological evolution are inherently difficult for these groups. Still, at least one other group of cyclic parthenogens, aphids, has been shown to undergo a very rapid adaptive radiation as evidenced by modelling bacterial endosymbiont divergence. Other groups with mixed breeding systems, such as protists and rotifers, appear to have many morphologically cryptic lineages, as well as some Cladocera [59]–[62]. Numerous candidate groups for recent radiations are apparent in Cladocera and Rotifera. The Ponto-Caspian for example, contains two potentially recent species flocks involving cyclic parthenogens from the order Onychopoda [63]. In *Daphnia*, several closely related forms found in neighbouring or connected waters could be the products of rapid radiations as well [64]. Recent radiations in cyclic parthenogens could have been overlooked because of the use of slowly evolving genetic markers [47], [48]. Our study and the recent evidence from other systems challenge the proposals that organisms with mixed breeding systems suffer a reduced speciation potential. Indeed, we argue that proposals for a weak speciation capacity in organisms with mixed breeding systems are also inconsistent with the antiquity of clades such as the Cladocera. If mixed breeding systems lead to weak speciation potentials, then macroevolutionary processes would likely replace such lineages with strictly sexual lineages [65]. No such breeding system conversion is known in cyclic parthenogens.

We have little direct knowledge of the mechanism of origination. We still know little about the role of hybridization in the radiation or why so many extreme forms exist in the pelagic zone of eutrophic waters. More work is also needed to assess the role of human activities (deforestation and agriculture) on the radiation, particularly as there is an association of lineages to nutrient status and intensity of predation in these lakes. Our study does reveal, however, that even under a presumed worst-case scenario for speciation, with mixed breeding systems and strong vagility and a relatively homogenous limnetic habitat, rapid radiations happen.

### Conclusions

We reject the hypothesis that morphotypes of *Eubosmina* lack morphological and genetical discreteness under sympatry. Instead, the results are consistent with the establishment of Holocene reproductive barriers as predicted by the detailed paleolimnological record. We observed the expected pattern of genetic differentiation with incomplete lineage sorting. We conclude that mixed breeding systems with weak sexual recruitment fail to preclude rapid radiations, but are instead, associated with some of the most rapid radiations known in animals.

## Methods

### Sample Collection

Bosminid taxonomy remains in a state of flux. We have therefore followed the latest taxonomy of *Eubosmina* with four species (*B. (E.) coregoni*, *B. (E.) longispina*, *B. (E.) longicornis*, *B. (E.) crassicornis*) and eleven subspecies (*B. (E.) coregoni coregoni*, *B. (E). c. gibbera*, *B. (E.) c. thersites*, *B. (E.) longispina longispina*, *B. (E.) l. reflexa*, *B. (E.) l. ruhei*, *B. (E.) longicornis longicornis*, *B. (E.) l. berolinensis*, *B. (E.) l. cederstroemi*, *B. (E.) l. kessleri*, *B. (E.) crassicornis*) based on morphological traits [29]. Our study includes seven members out of those eleven subspecies belonging to three species and we use their subspecies names only and treated them as morphotypes. The studied lakes were: Vänern (Sweden), N 27.58°47.7′, E 12°41.4′, with *longispina* (ls) and *coregoni* (cor) morphotypes, sampled on June 16, 2004; Stora Färgen (Sweden), N 56°57.7′, E 13°20.7′, with *longispina*, *cederstroemi* (ced) and *longicornis* (lc) morphotypes sampled on June 29, 2004; Ragnerudsjön (Sweden), N 58°37.6′, E 12°5.6′, with *longispina*, *kessleri* (kes) and *longicornis* morphotypes sampled on September 25, 2002; Fleesensee (Germany), N53°29′, E 12°28′, with *berolinensis* (ber), *coregoni* and *gibbera* (gibb) morphotypes, sampled on May 8, 2004. These lakes were chosen based on coexistence of morphotypes. We have a record of coexistence of *longispina* and *coregoni* morphotypes at our sampling site in Vänern Lake from 2002 and historic samples documenting the coexistence of *longispina*, *cederstroemi* and *longicornis* morphotypes as far back as in 1933 in Lake Stora Färgen. Specimens were preserved in 96% ethanol.

### DNA extraction, PCR, sequencing and cloning

We extracted nucleic acid from single adult individuals by incubation in 15–20 µl of Quick-Extract (Epicentre) for 2 hours at 65°C followed by 10 min at 98°C. We performed PCR in a 30 µl total reaction using 3 µl of extract, 3 µl of 10× PCR buffer, 0.6 µl of each of 10 mM dNTPs solution, 0.9 µl of each 10 µM primer and 0.6 unit of Taq polymerase with 1.5 mM MgCl_2_. The PCR conditions were 94°C for 30 s, 50°C for 30 s, 72°C for 1 min, 20 sec for 40 cycles, followed by 72°C for 7 min for the complete mitochondrially-encoded NADH dehydrogenase subunit 2 (ND2) gene sequences. The PCR conditions for the nuclear-encoded Heat Shock Protein 90 (HSP90) gene sequences were the same except for annealing time, which was just 1 minute. We designed the following primers: MetR4 (5′-GCTTCAGCTTCGGCCATCCTGTCAG-3′), R1 (5′-AATAAACTTAAACTGGTAGAGCAGGTCCC-3′) for *ND2* and HSP-R1 (5′-TCACCAACGTCTTCGACTTTGGGTTCC-3′), HSP F2 (5′- ACAAGTTGGACAGTGGCAAGGAGCTG-3′) for *HSP90*. PCR products were sent for sequencing to Genaissance pharmaceuticals (Connecticut, USA). We cloned apparent heterozygous PCR products with the TOPO TA Cloning® Kit for Sequencing (Invitrogen™) to obtain both alleles of *HSP90* gene. Clones were sequenced until both alleles could be differentiated. The average number of cloned sequences per one heterozygous PCR product was four.

### DNA sequences alignments and analyses

We assembled and edited sequences using Sequencher 4.2. (Gene Codes Corporation) and manually aligned both *ND2* and *HSP90*. Sequences were deposited in Genbank under the following accession numbers GU249620-GU250313.

We have analyzed following numbers of haplotypes and morphotypes on the genes under this study: Lake Vänern: *ND2*: 33 haplotypes (ls 39, cor 40), *HSP*: 28 haplotypes (ls 41, cor 40); Lake Stora Färgen *ND2*: 25 haplotypes (ced 35, ls 26, lc 7), *HSP*: 22 haplotypes (ced 27, ls 22, lc 7); Lake Ragnerudsjön *ND2*: 21 haplotypes (ls 19, kes 23, lc 2), *HSP*: 9 haplotypes (ls 23, kes 26, lc 3); Fleesensee *ND2*: 13 haplotypes (ber 19, cor 13, gibb 17), *HSP*: 18 haplotypes (ber 18, cor 2, gibb 18).

We used the TCS 1.21 program [66] to estimate haplotype networks with connections that have a 95% probability of being the most parsimonious. Indels were treated as a fifth character for the putative introns of *HSP90* sequences. We calculated genetic differentiation (F_ST_ and Φ_ST_ values) in ARLEQUIN 3.01 [67], where F_ST_'s are based on the frequencies of haplotypes and Φ_ST_'s are based on the genetic distances among haplotypes. We tested statistical significance by a permutation procedure. To calculate F_ST_'s we pooled all unshared haplotypes into one. We used the genealogical sorting index (*gsi*) to quantify the genealogical exclusivity of coexisting morphotypes [68]. The *gsi* is an index that tracks genealogical differentiation in young species from polyphyly (*gsi* = 0) to monophyly (*gsi* = 1). The input trees were calculated using PHYML 3.0 [69] with a GTR substitution model, proportion of invariable sites and among site rate parameters estimated from the data. We used the most complex model but note that neither substitution model choice nor over-parameterization has much effect on analyses with very closely related sequences. Trees were outgroup rooted using specimens from the established sister group, *Eubosmina* sp. from North America. Significance of the *gsi* was calculated by permutation (10000 replicates). Because unbalanced comparisons can change the results, we excluded comparisons of the morphotypes with less than seven specimens (see Table 1).

### Geometric morphometry

We carried out pairwise geometric morphometric comparisons between coexisting morphotypes (collected from the same planktonic sample) of adult *Eubosmina* to examine their discreteness based on carapace shape. We compared the shapes of the dorsal margin of the carapace from the point of the upper insertion of the antennules to the posterior point where the carapace opens (Fig. 2). The two endpoints represent homologous landmarks. The antennules and the mucro that are the main structures used to classify most of the morphotypes were not part of the morphometric analyses.

We digitized the outline of the dorsal margins of the carapaces using tpsDig [70] of following numbers of specimens: Lake Vänern: ls 22, cor 22; Lake Stora Färgen ced 22, ls 18, lc 8; Lake Ragnerudsjön: ls 18, kes 21, lc 6; Fleesensee: ber 5, cor 7, gibb 5.

We then aligned the digitized specimens in Chainman [71], reducing the curve to twenty landmarks. The semi-landmark alignment involved a generalized least squares procrustes superimposition followed by a distance minimizing procedure to mathematically remove differences between the curves attributable to random positioning of points along the curve [72]–[74]. We used Goodall's F-test [75] with p-values determined by 900 random permutations of the data in TwoGroup6h [71], [76] to test if different morphs within each lake have statistically significant pairwise differences in the average shape. We calculated Procrustes distances (the standardized measure of the distance between shapes used to e.g. test whether the distance between one pair of samples differs from the distance between another pair of analyzed samples) in TwoGroup6h to test how the magnitudes of differences between the mean forms vary among the morphs when there are meaningful differences in shapes. We also applied Canonical Variates Analysis (CVA) in CVAGen6 [71], [76] to determine if we could assign specimens to morphotype based purely on the measured shape of the specimens when considering all morphotypes simultaneously, and we estimated the rate of correct assignments with a jackknife-test of assignments based on the CVA axes. In the jackknife procedure, each specimen is omitted in turn from the calculation of the CV axes, and then assigned to a morphospecies based on those axes. The jackknife rate of correct assignments is a reasonable estimate of how well the CVA would perform in assigning newly acquired specimens to the correct morphotype or morphotype discreteness.

## References

[1] Mayr E (1963). Animal species and evolution..

[2] Coyne J, Orr H (2004). Speciation..

[3] Barraclough TG, Birky CW, Burt A (2003). Diversification in sexual and asexual organisms.. Evolution.

[4] Bell G, Butlin R, Bridle J, Schluter D (2009). The poverty of the protists.. Speciation and patterns of diversity.

[5] Fontaneto D, Herniou EA, Boschetti C, Caprioli M, Melone G (2007). Independently evolving species in asexual bdelloid rotifers.. PLoS Biol.

[6] De Meester L, Gomez A, Okamura B, Schwenk K (2002). The monopolization hypothesis and the dispersal-gene flow paradox in aquatic organisms.. Acta Oecol.

[7] Morran L, Parmenter M, Phillips P (2009). Mutation load and rapid adaptation favour outcrossing over self-fertilization.. Nature.

[8] Lynch M (1984). The limits to life-history evolution in *Daphnia*.. Evolution.

[9] Lynch M (1985). Speciation in Cladocera.. Verh Internat Verein Limnol.

[10] Peccoud J, Simon JC, McLaughlin HJ, Moran NA (2009). Post-Pleistocene radiation of the pea aphid complex revealed by rapidly evolving endosymbionts.. Proc Natl Acad Sci U S A.

[11] Frey DG (1958). The late-glacial Cladoceran fauna of a small lake.. Arch Hydrobiol.

[12] Frey DG (1960). The ecological significance of Cladocera remains in lake sediments.. Ecology.

[13] Frey DG (1962). Cladocera from the Eemian interglacial of Denmark.. J Paleontology.

[14] Frey DG (1962). Paleolimnology of freshwater lakes.. Japan Soc Limnology.

[15] Goulden CE (1964). The history of the cladoceran fauna of Esthwaite Water (England) and its limnological significance.. Arch Hydrobiol.

[16] Shan RK-C (1969). Life cycle of a chydorid cladoceran, *Pleuroxus denticulatus* Birge.. Hydrobiologia.

[17] Hofmann W (1977). *Bosmina* (*Eubosmina*) populations of the Groesseer Segebergersee during late glacial and postglacial times.. Arch Hydrobiol.

[18] Hofmann W (1978). *Bosmina* (*Eubosmina*) populations of the Grosserer Ploenersee and Schoehsee lakes during late-glacial and postglacial times.. Pol Arch Hydrobiol.

[19] Nauwerck A (1978). *Bosmina-obtusirostris* Sars in Lake Latnjajaure, Swedish Lappland.. Arch Hydrobiol.

[20] Hofmann W (1984). Postglacial morphological variation in *Bosmina longispina* Leydig (Crustacea, Cladocera) from the Grosser Ploner See (north Germany) and its taxonomic implications.. Z zool Syst Evolut-forsch.

[21] Hofmann W (1984). Morphological variation in a late glacial population of *Bosmina longispina* Leydig (Crustacea, Cladocera) from the Lobsigensee (Swiss Plateau). Studies in the Late Quaternary of Lobsigensee 9.. Schweiz Z Hydrol.

[22] Hofmann W (1986). On the relationship between *Bosmina* taxa coregoni and thersites (Cladocera), as indicated by subfossil remains.. Hydrobiologia.

[23] Hofmann W (1987). The Late Pleistocene/Holocene and Recent *Bosmina* (*Eubosmina*) fauna (Crustacea: Cladocera) of the pre-alpine Starnberger See (FRG).. J Plankton Res.

[24] Hofmann W (1991). The Late-Glacial/Holocene *Bosmina* (*Eubosmina*) fauna of Lake Constance (Untersee) (F.R.G.): Traces of introgressive hybridization.. Hydrobiologia.

[25] Nauwerck A (1991). *Bosmina* changes in the sediments of Lake Mondsee.. Hydrobiologia.

[26] Guenther J, Lieder U (1993). Postglacial succession in the subgenus *Eubosmina* (Crustacea: Cladocera) in the region of the Unterhavel River (near Berlin, Germany): Type changes or species immigration?. Int Revue ges Hydrobiol.

[27] Hofmann W (1993). Late-Glacial/Holocene changes of the climatic and trophic conditions in three Eifel maar lakes, as indicated by faunal remains. I. Cladocera.. Lecture Notes in Earth Sciences.

[28] Hofmann W (1994). Morphologische variation der plankton-Cladocere *Bosmina* (*Eubosmina*) im Selenter See.. Faun-ökol Mitt.

[29] Lieder U (1996). Crustacea.. Cladocera, Bosminidae Süβwasserfauna von Mitteleuropa.

[30] Haney RA, Taylor DJ (2003). Testing paleolimnological predictions with molecular data: the origins of Holarctic *Eubosmina*.. J Evol Biol.

[31] Maemets A, Timm M, Noges T (1996). Zooplankton of Lake Peipsi-Pihkva in 1909-1987.. Hydrobiologia.

[32] Hellsten ME, Stenson JAE (1995). Cyclomorphosis in a population of *Bosmina coregoni*.. Hydrobiologia.

[33] Nilssen JP, Halvorsen G, Melaen JG (1980). Seasonal divergence of *Bosmina* morphs.. Int Revue ges Hydrobiol.

[34] Kerfoot WC (2006). The Baltic *Eubosmina* radiation: sensitivity to invertebrate predators (induction) and observations on genetic differences.. Arch Hydrobiol.

[35] Johnsen GF, Raddum GG (1987). A morphological study of two populations of *Bosmina longispina* exposed to different predation.. J Plankton Res.

[36] Hellsten ME, Lagergren R, Stenson J (1999). Can extreme morphology in *Bosmina* reduce predation risk from *Leptodora*? An experimental test.. Oecologia.

[37] Lagergren R, Lord H, Stenson JAE (2000). Influence of temperature on hydrodynamic costs of morphological defences in zooplankton: experiments on models of *Eubosmina* (Cladocera).. Func Ecol.

[38] Lagergren R, Stenson JAE (2000). Chemical cues from the invertebrate predator *Leptodora kindtii* affect the development of cyclomorphic traits in *Eubosmina coregoni gibbera*.. J Plankton Res.

[39] Flössner D, Verlag GF (1972). Kiemen- und Blattfusser, Branchiopoda, Fischlause, Branchiura..

[40] Kerfoot WC (1981). Long-term replacement cycles in cladoceran communities: a history of predation.. Ecology.

[41] Hofmann W (1987). Cladocera in space and time - Analysis of lake sediments.. Hydrobiologia.

[42] Nauwerck A (1991). The History of the Genus *Eubosmina* in Lake Mondsee (Upper Austria).. Hydrobiologia.

[43] Lilljeborg W (1901). Cladocera Sueciae.. Nova Acta reg soc sci Upsala ser 3.

[44] Hellsten ME, Sundberg P (2000). Genetic variation in two sympatric European populations of *Bosmina* spp. (Cladocera) tested with RAPD markers.. Hydrobiologia.

[45] Barluenga M, Stölting KN, Salzburger W, Muschick M, Meyer A (2006). Sympatric speciation in Nicaraguan crater lake cichlid fish.. Nature.

[46] Omland KE, Baker JM, Peters JL (2006). Genetic signatures of intermediate divergence: population history of Old and New World Holarctic ravens (*Corvus corax*).. Mol Ecol.

[47] Ishida S, Taylor DJ (2007). Quaternary diversification in a sexual Holarctic zooplankter, Daphnia galeata.. Mol Ecol.

[48] McCormack J, Bowen B, Smith T (2008). Integrating paleoecology and genetics of bird populations in two sky island archipelagos.. BMC biology.

[49] Elmer KR, Lehtonen TK, Kautt AF, Harrod C, Meyer A (2010). Rapid sympatric ecological differentiation of crater lake cichlid fishes within historic times.. BMC biology.

[50] Zink R (2002). A new perspective on the evolutionary history of Darwin's finches.. The Auk.

[51] Futuyma D (2008). Ecology, speciation, and adaptive radiation: the long view.. Evolution.

[52] Mallet J (2008). Hybridization, ecological races and the nature of species: empirical evidence for the ease of speciation.. Phil Trans R Soc London B.

[53] Knowles LL, Carstens BC (2007). Delimiting species without monophyletic gene trees.. Syst Biol.

[54] Shaffer HB, Thomson RC (2007). Delimiting species in recent radiations.. Syst Biol.

[55] Ritz MS, Millar C, Miller GD, Phillips RA, Ryan P (2008). Phylogeography of the southern skua complex - rapid colonization of the Southern hemisphere during a glacial period and reticulate evolution.. Mol Phylogenet Evol.

[56] Taylor DJ, Hebert PDN (1992). *Daphnia galeata mendotae* as a cryptic species complex with interspecific hybrids.. Limnol Oceanogr.

[57] Little TJ, Demelo R, Taylor DJ, Hebert PDN (1997). Genetic characterization of an Arctic zooplankter insights into geographic polyploidy.. Proc R Soc Lond Ser B.

[58] Adamowicz SJ, Gregory TR, Marinone MC, Hebert PDN (2002). New insights into the distribution of polyploid *Daphnia*: the Holarctic revisited and Argentina explored.. Mol Ecol.

[59] Adamowicz SJ, Hebert PDN, Marinone MC (2004). Species diversity and endemism in the *Daphnia* of Argentina: a genetic investigation.. Zool Jour Linn Soc.

[60] Belyaeva M, Taylor DJ (2009). Cryptic species within the *Chydorus sphaericus* species complex (Crustacea: Cladocera) revealed by molecular markers and sexual stage morphology.. Mol Phylogenet Evol.

[61] Fontaneto D, Kaya M, Herniou EA, Barraclough TG (2009). Extreme levels of hidden diversity in microscopic animals (Rotifera) revealed by DNA taxonomy.. Mol Phylogenet Evol.

[62] Stoeck T, Behnke A, Christen R, Amaral-Zettler L, Rodriguez-Mora MJ (2009). Massively parallel tag sequencing reveals the complexity of anaerobic marine protistan communities.. BMC Biology.

[63] Cristescu MEA, Hebert PDN (2002). Phylogeny and adaptive radiation in the Onychopoda (Crustacea, Cladocera): evidence from multiple gene sequences.. J Evol Biol.

[64] Fisk D, Latta L, Knapp R, Pfrender M (2007). Rapid evolution in response to introduced predators I: rates and patterns of morphological and life-history trait divergence.. BMC Evolutionary Biology.

[65] Taylor DJ, Crease TJ, Brown WM (1999). Phylogenetic evidence for a single long-lived clade of crustacean cyclic parthenogens and its implications far the evolution of sex.. Proc R Soc Lond Ser B.

[66] Clement M, Posada D, Crandall K (2000). TCS: a computer program to estimate gene genealogies.. Mol Ecol.

[67] Excoffier L, Laval G, Schneider S (2005). Arlequin (version 3.0): an integrated software package for population genetics data analysis.. Evolutionary Bioinformatics online.

[68] Cummings M, Neel M, Shaw K (2008). A genealogical approach to quantifying lineage divergence.. Evolution.

[69] Guindon S, Gascuel O (2003). A simple, fast, and accurate algorithm to estimate large phylogenies by maximum likelihood.. Syst Biol.

[70] Rohlf F (2004).

[71] Sheets HD (2005). IMP series software..

[72] Bookstein FL, Mardia KV, Gill CA, Dryden IL (1996). Applying landmark methods to biological outline data.. Image fusion and shape variability.

[73] Sampson PD, Bookstein FL, Sheehan H, Bolson EL, Marcus LF, Corti M, Loy A, Naylor GJP, Slice DE (1996). Eigenshape analysis of left ventricular outlines from contrast ventriculograms.. Advances in Morphometrics.

[74] Bookstein FL (1997). Landmark methods for forms without landmarks: morphometrics of group differences in outline shape.. Medical Image Analysis.

[75] Goodall C (1991). Procrustes methods in the statistical analysis of shape. Journal of the Royal Statistical Society.. Series B (Methodological).

[76] Zelditch ML, Swiderski DL, Sheets DH, Fink WL (2004). Geometric morphometrics for biologists: a primer.. Elsevier.

